# Influence of β-Cyclodextrin Methylation on Host-Guest Complex Stability: A Theoretical Study of Intra- and Intermolecular Interactions as Well as Host Dimer Formation

**DOI:** 10.3390/molecules28062625

**Published:** 2023-03-14

**Authors:** Niklas Geue, Jackson J. Alcázar, Paola R. Campodónico

**Affiliations:** 1 Michael Barber Centre for Collaborative Mass Spectrometry, Manchester Institute of Biotechnology, Department of Chemistry, The University of Manchester, 131 Princess Street, Manchester M1 7DN, UK; 2 Centro de Química Médica, Facultad de Medicina Clínica Alemana, Universidad del Desarrollo, Santiago 7780272, Chile

**Keywords:** non-covalent interactions, density functional theory, methylated cyclodextrins, dimethyl-β-cyclodextrin, hydrogen bond

## Abstract

Understanding the non-covalent interactions in host-guest complexes is crucial to their stability, design and applications. Here, we use density functional theory to compare the ability of β-cyclodextrin (**β-CD**) and heptakis(2,6-di-O-methyl)-β-cyclodextrin (**DM-β-CD**) to encapsulate the model guest phenol. For both macrocycles, we quantify the intramolecular interactions before and after the formation of the complex, as well as the intermolecular host-guest and host-host dimer interactions. These are individually classified as van der Waals interactions or hydrogen bonds, respectively. The results show a stronger intramolecular binding energy of **β-CD**, with the absolute difference being −5.53 kcal/mol relative to **DM-β-CD**. Consequently, the intermolecular interactions of both cyclodextrins with phenol are affected, such that the free binding energy calculated for the **DM-β-CD**/phenol complex (−5.23 kcal/mol) is ≈50% more negative than for the complex with **β-CD** (−2.62 kcal/mol). The latter is in excellent agreement with the experimental data (−2.69 kcal/mol), which validates the level of theory (B97-3c) used. Taken together, the methylation of **β-CD** increases the stability of the host-guest complex with the here studied guest phenol through stronger van der Waals interactions and hydrogen bonds. We attribute this to the disruption of the hydrogen bond network in the primary face of **β-CD** upon methylation, which influences the flexibility of the host toward the guest as well as the strength of the intermolecular interactions. Our work provides fundamental insights into the impact of different non-covalent interactions on host-guest stability, and we suggest that this theoretical framework can be adapted to other host-guest complexes to evaluate and quantify their non-covalent interactions.

## 1. Introduction

**Figure 1:**
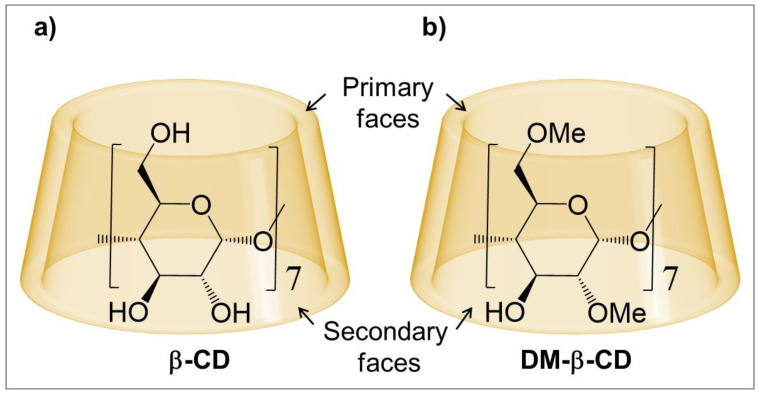
Schematic of the macrocycles: (**a**) β-Cyclodextrin (**β-CD**) and (**b**) Heptakis(2,6-di-O-methyl)-β-cyclodextrin (**DM-β-CD**).

Cyclodextrins (CDs) consist of circularly connected glucose units, joined by α-1,4 glycosidic bonds (Figure 1a), and are well-known to encapsulate small molecules forming host-guest complexes [1,2,3,4]. The general structure of CDs is toroidal, with two openings (“faces”), and the primary and secondary alcohol groups are located at the smaller and larger opening, respectively (Figure 1). The inside of CDs is hydrophilic, but significantly less hydrophilic than the common aqueous environment, and hence both apolar and polar guests can be hosted in their cavity.

Host-guest complexes with CDs have attracted broad interest in the last decades, driven by myriad applications, including drug delivery [5,6,7,8], food science [9,10], sensing [11,12], petroleum chemistry [13] and pollutant removal [14,15]. These inclusion complexes have been studied and extensively characterized by a range of analytical techniques [16]; however, decoupling their non-covalent interactions on a fundamental level is often difficult with experimental solution phase techniques, where all non-covalent interactions contribute simultaneously [17]. Interactions in CD inclusion complexes have been extensively modelled with computational techniques [18], predominantly with molecular dynamics simulations [19] and density functional theory (DFT) [20]. The latter was shown to be ideally suited to model the non-covalent interactions of CD complexes with a high level of accuracy [20], and recently a method to estimate the energies and nature of non-covalent interactions from the density of critical bonding points has been reported [21]. We suggest that this methodology can also be applied to CD-based host-guest complexes.

In CDs, the number of glucose units controls the size of their cavity, and possibly the most prevalent cyclodextrin is β-cyclodextrin (**β-CD**), which involves seven glucopyranoside monomers (Figure 1a). One major drawback of **β-CD** is its low solubility in water [22]; however, a significant increase is observed when substituting the hydroxyl groups with neutral or ionic substituents [23], e.g., with methyl groups [24]. Methylation of CDs not only increases their solubility, but also enhances the stability of their host-guest complexes [25,26,27,28,29,30,31,32,33,34], for example up to ≈1.2 kcal/mol for the guests methyl orange and 8-anilino-1-naphthalenesulfonate [31,32].

Methylated (β)-CDs have been proposed for applications in polymer chemistry [35,36,37], soil decontamination [38,39], batteries [40], pharmaceutics [41] and as chiral selectors in electrophoresis [42,43], and for most of these the formation of highly stable host-guest complexes is essential. Hence, a precise understanding of their non-covalent interactions is warranted in order to tune their inclusion complex properties. Previously, Wenz and Höfler investigated the effect of methylation on the energetics of **β-CD** host-guest complexes using isothermal titration microcalorimetry, showing that binding free energies and enthalpies significantly depend on the degree and place of **β-CD** methylation [33,44]. More precisely, Wenz found that methylation in the primary face enhances the binding constant, whereas the opposite was observed for methylation in the secondary face [33].

Applying DFT, we extend the work by Wenz to reveal the impact of methylation on the hosting ability of **β-CD,** using the methylated cyclodextrin heptakis(2,6-di-O-methyl)-β-CD, often referred to as dimethyl-β-cyclodextrin (**DM-β-CD**, Figure 1b). Compared to **β-CD**, the primary and one of the two secondary hydroxy (-OH) groups are substituted for methoxy (-OCH_3_) groups in each of the monomers of **DM-β-CD**. This macrocycle is therefore ideally suited to assess the methylation in both faces of **β-CD**, while still one secondary -OH group remains as an internal comparison to **β-CD**. In this context, the characterization and comparison of the different non-covalent interactions in the CD inclusion complexes is possible, in particular (i) intramolecular interactions within the CD hosts to determine the effect of the -OH and -OCH_3_ groups, (ii) intermolecular interactions between the CD hosts and the guest (in this study phenol) to compare the complex stability in terms of van der Waals (vdW) interactions, hydrogen bonds (HBs) and binding free energy, and (iii) the formation of host dimers to estimate their contribution to the underestimation of the observed binding constant.

## 2. Results and Discussion

### 2.1. Intramolecular Interactions within the β-CD and DM-β-CD Macrocycles

**Figure 2:**
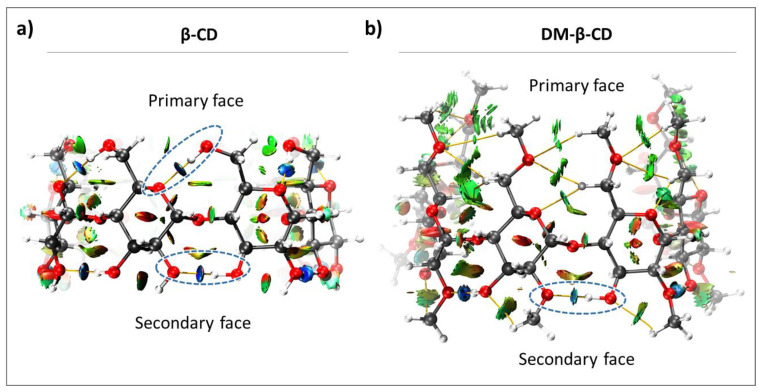
Color-filled RDG isosurface for intramolecular interactions within (**a**) **β-CD** and (**b**) **DM-β-CD**. The different types of interaction regions are highlighted, blue: attractive interactions (hydrogen bonds), green: slightly attractive vdW interactions, red: repulsive steric interactions. Hydrogen bonds are additionally marked with blue, dashed ellipsoids.

The intramolecular interactions in **β-CD** and **DM-β-CD** were evaluated, and since both constitute of seven monomeric units, we investigated the seven intramolecular host interactions occurring between the monomers on both faces. As shown in Figure 2, **β-CD** exhibits intramolecular HBs on both faces, while **DM-β-CD** only has HBs on the secondary face. This leads to a higher degree of freedom of the primary face in **DM-β-CD,** compared to the highly rigid **β-CD** [45]. The only significant contribution to the interactions in the primary face of **DM-β-CD** is vdW interactions (green regions, Figure 2b), whose energies were estimated together with those of the HBs in the primary face (Table 1).

The individual HB interaction energies, estimated for each face, are largely constant in **β-CD**, and this can be attributed to its high symmetry and structural rigidity (Table 1). In contrast, the individual intramolecular interactions of **DM-β-CD** show more fluctuations, particularly in the secondary face (standard error in Table 1). This is due to a substantial loss of symmetry/rigidity between the monomers that constitute **DM-β-CD**, caused by the absence of HBs at the primary face and the electronic repulsions experienced by the -OCH_3_ groups. The absolute average energies of the intramolecular interactions were significantly different for both macrocycles, with **β-CD** having stronger intramolecular interaction on both faces (Table 1). The primary face of **β-CD** shows a 5.53 kcal/mol higher averaged intramolecular interaction energy than **DM-β-CD**, while the intramolecular interactions on the secondary face yielded a smaller difference of 1.55 kcal/mol between both hosts.

Taken together, the intramolecular interactions within **DM-β-CD** are weaker than for **β-CD**, due to methylation and the absence of intramolecular HBs in the primary face of **DM**-**β-CD**. Hence, **DM-β-CD** is less rigid than **β-CD** and is expected to be more flexible in encapsulating guest molecules.

### 2.2. Intermolecular Interactions between β-CD/DM-β-CD and the Model Guest Phenol

After evaluating the non-covalent interactions within both CD hosts, we investigated the intermolecular interactions between **β-CD**/**DM-β-CD** and the guest phenol occurring in host-guest complexes of the stoichiometry 1:1 [46]. We chose phenol for this purpose as it is a simple molecule constituted of a hydrophobic group (phenyl moiety) and a hydrogen bond acceptor and donor (hydroxyl group). This makes it possible to reliably evaluate both vdW interactions and hydrogen bonds occurring with the CD hosts [26].

**Figure 3:**
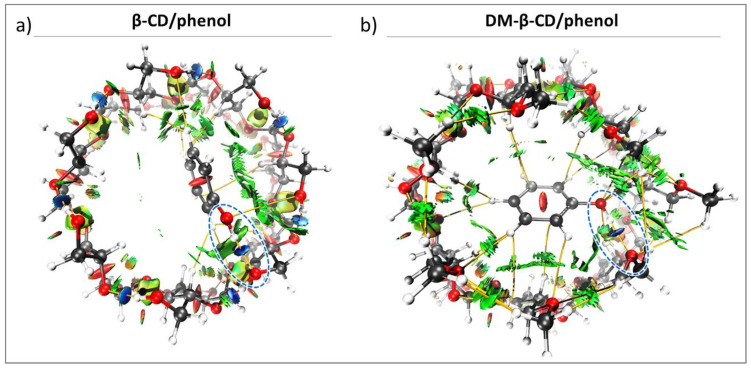
Color-filled RDG isosurface for intermolecular host-guest interactions between (**a**) **β-CD**/phenol and (**b**) **DM-β-CD**/phenol. The different types of interaction regions are highlighted in different colors; blue: attractive interactions (hydrogen bonds), green: slightly attractive vdW interactions, red: repulsive steric interactions. Hydrogen bonds between the host and phenol are additionally marked with blue, dashed ellipsoids.

The DFT optimized structure of the **β-CD**/phenol inclusion complex shows a docked host-guest conformation, with most of the phenol being included within the cavity. The hydroxyl group interacts favourably with the portals of the primary face of **β-CD** (via OH-OH), and this is in agreement with previous results obtained from NMR spectroscopy [46]. Similar results were found for the **DM-β-CD**/phenol complex, where the guest hydroxyl group interacts with the primary face of **DM-β-CD** (via OH-OCH_3_). Because the hydroxyl group of the phenol interacts with the same side of both CDs (Figure 3), the effect of the CD methylation at the primary face on the stability of the inclusion complex can be directly compared (Table 2). The results show that the intermolecular HB energy of **β-CD** (between the host and guest hydroxyl groups) was −4.99 kcal/mol, while the OH-OCH_3_ interaction in the **DM-β-CD**/phenol complex was stronger (−7.31 kcal/mol). This difference can be rationalized with the intramolecular interactions in the CD hosts, specifically the intramolecular interaction of the host oxygen that interacts with the hydroxyl group of phenol. Here, the **β-CD**/phenol complex presents with a strong HB of −5.38 kcal/mol, which is highly similar to the free **β-CD** as discussed above (Table 1). For the **DM-β-CD**/phenol complex, the strong and attractive intramolecular interaction does not occur (+0.17 kcal/mol, Table 2), and this allows the -OCH_3_ groups to form strong OH-OCH_3_ HBs with phenol.

For the **β-CD**/phenol and **DM-β-CD**/phenol complexes, the interaction energies (−14.96 kcal/mol and −18.27 kcal/mol, respectively) are mainly governed by vdW interactions (ca. 70%, Table 2), as previously reported for similar complexes [28,34,47]. The estimated ΔΕ_vdW_ for the **β-CD**/phenol complex is −11.40 kcal/mol, while for the **DM-β-CD**/phenol complex, the energy is −14.67 kcal/mol. This difference can be explained by the greater flexibility that **DM-β-CD**/phenol possesses by having a weaker network of intramolecular interactions compared to **β-CD** (Table 1) [33,48]. This is in contrast to the rationalization suggested by Wenz, who assumed the absence of an intramolecular hydrogen network in the primary face of **β-CD** due to the too long distance between the hydroxyl groups, and hence that methylation there had no major influence on the rigidity of the host [33]. In our work, we found intramolecular HBs in the primary face of **β-CD** (discussed above) and a higher flexibility when these were absent in **DM-β-CD**. This apparently allows **DM-β-CD** to organize itself and adapt a favorable cavity for phenol, changing the position of the guest within the complex to a conformation with stronger vdW interactions (Figure 3, green).

Finally, ΔG_calc_ was calculated for both complexes (Table 2). The **DM-β-CD**/phenol complex has a binding energy of −5.23 kcal/mol, whereas the one of the **β-CD**/phenol complex is −2.62 kcal/mol. The latter is in excellent agreement with calorimetric data previously reported (−2.69 kcal/mol) [26], and this validates the level of theory used in this work. The absolute difference in binding energy (2.61 kcal/mol, ≈50%) between both complexes supports the points discussed above, and suggests that **DM-β-CD** is a better host than **β-CD** for the guest phenol. For the same reasons, we also anticipate that **DM-β-CD**, compared to **β-CD**, is better suited to form stable host-guest complexes with alcohols in general.

### 2.3. Dimers of the Macrocycles β-CD and DM-β-CD

A detailed knowledge of the species present in assays with macrocycles such as CDs is important for experimentally determining host-guest binding constants of a given complex. The latter depends on the concentration of the free host and hence the formation of dimers (or even oligomers) leads to an absolute concentration decrease, resulting in an underestimation of the binding constant. Thus, the stability of the **β-CD** and **DM-β-CD**-based dimers is linked to the differences in their observed host-guest binding constants and should hence be considered when discussing host-guest complex stability.

**Figure 4:**
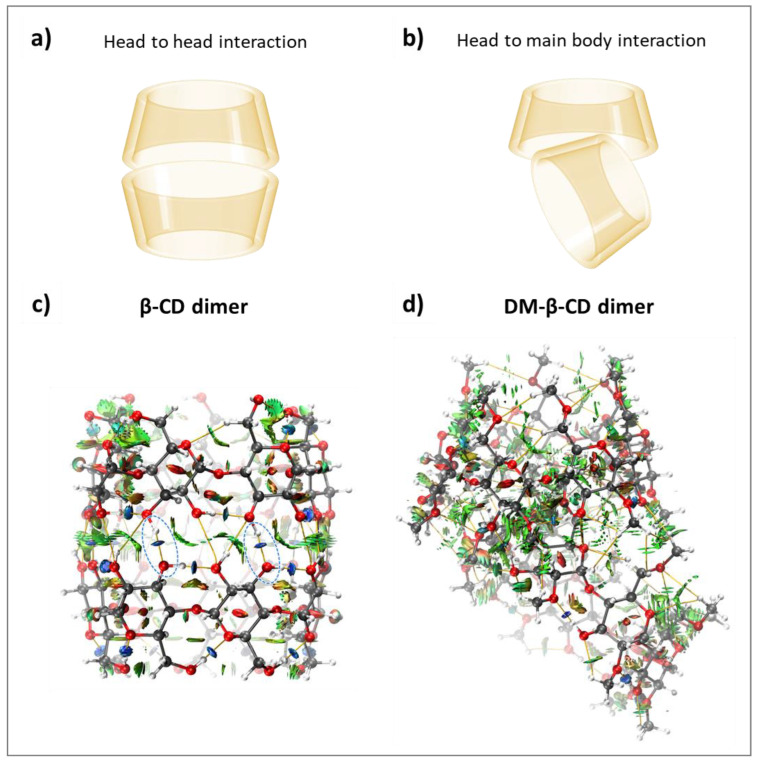
Schematic of (**a**) head-to-head interactions and (**b**) head to main body interactions of **β-CD** and **DM-β-CD** dimers, respectively. Color-filled RDG isosurface for intermolecular host-host interactions between (**c**) **β-CD** dimers and (**d**) **DM-β-CD** dimers. The different types of interaction regions are highlighted in different colors, blue: attractive interactions (hydrogen bonds), green: slightly attractive vdW interactions, red: repulsive steric interactions. Hydrogen bonds are additionally marked with blue, dashed ellipsoids.

The dimer formation of CDs was previously reported in the literature, and the favoured host-host conformation of **β-CD** is head-to-head (Figure 4a) [49,50]. For both macrocycles, inter- and intramolecular HBs as well as vdW interactions were quantified (Table 3). The **β-CD** dimer was found to be more stable (ΔΕ_int_ = −53.88 kcal/mol) than the **DM-β-CD** dimer (ΔΕ_int_ = −41.65 kcal/mol), and the comparison of ΔG_calc_ for both dimers was not possible, as the value for **DM-β-CD** could not be determined. The large ΔΕ_int_ difference can be rationalized with the different dimer conformations (head-to-head for **β-CD** and head to main body for **DM-β-CD**, Figure 4a,b) and the occurrence of intermolecular hydrogen bonds for **β-CD** (−5.33 kcal/mol averaged over each monomer, Table 3, Figure 4c). In contrast, the head to main body dimer of **DM-β-CD** lacks intermolecular HB interactions (Figure 4d). This is also quantitatively reflected in the electrostatic interaction energies of both dimers, where the **β-CD** dimer yields ΔΕ_ele_ = −46.40 kcal/mol and the **DM-β-CD** dimer ΔΕ_ele_ = −13.28 kcal/mol (Table 3). Noteworthy is also that the averaged individual intramolecular interaction energy in the **β-CD** dimer (−5.22 kcal/mol, Table 3) is significantly weaker compared to the one of the secondary face of the **β-CD** monomer (−5.86 kcal/mol, Table 1), which once more highlights the interplay between intramolecular and intermolecular interactions.

## 3. Methods

### 3.1. Preparation of Initial Structures and Geometry Optimization

The initial structures of **β-CD** and **DM-β-CD** were obtained from X-ray crystallography data [51,52]. These were used as inputs for electronic structure optimization using density functional theory (method-basis set B97-3c) [53,54], under the TightSCF convergence protocol established by default in the ORCA program package (Program Version 5.0.3) [55]. The method-basis set B97-3c was chosen because it is a low-cost and reliable level of theory [54], suitable for evaluating large systems such as cyclodextrin dimers. The accuracy was validated with the comparison of the free binding energy of **β-CD**/phenol with the experimental literature data (Table 2), and hence other levels of theory were not tested. The local energy minimum for each electronic structure was confirmed as all vibrational modes presented positive magnitudes.

The initial interaction conformation of the host-host and host-guest complexes were docked from the Autodock program (Version 4.2) [56] using Merz-Kollmann (MK) charges [57], for a 1:1 complex stoichiometry ratio. The MK charges were calculated from the optimized structures of the monomers using the Multiwfn program (Version 3.8) [58]. Docked complexes were optimized as described above for **β-CD** and **DM-β-CD**. Optimized coordinates and minimum energies of **β-CD**, **DM-β-CD** and phenol, as well as the corresponding host-guest complexes and host-host dimers, can be found in the supplementary dataset for both gas phase and aqueous phase calculations.

### 3.2. Noncovalent Interaction (NCI) Analysis

The NCI analysis was carried out by the reduced density gradient (RDG) method as described by Yang et al. [59], and as implemented in the Multiwfn program [58]. The electron densities (*ρ*) of the monomers and complexes were mapped according to Equation (1), as a function of the sign of the second largest eigenvalue of the Hessian matrix of electron density (sign(λ_2_)) multiplied by *ρ*, to distinguish between different types of noncovalent interactions at the critical bond points obtained from Bader’s AIM theory (Figure S1 for **β-CD/DM-β-CD** including the description of the color scale and Figure S2 for the **β-CD/DM-β-CD** complexes with phenol as well as the dimers of **β-CD** and **DM-β-CD**, respectively) [60]. For all cases, the chosen isovalue was 0.6, adequate for constructing the RDG isosurface and to reveal all the weak interactions involved in a 3D space.

(1) $$\mathrm{RDG}\;\left(r\right)=\frac{1}{{2(3\pi }^{2}{)}^{1/3}}\frac{\left|\nabla \rho (r)\right|}{{\rho (r)}^{4/3}}$$

### 3.3. Calculation of the Individual Non-Covalent Interactions Energies

The energies of the non-covalent interactions involved in the monomers and the different dimers/host-guest complexes were calculated as described by Lu et al. [21]. Each of the structures optimized with the B97-3c level of theory underwent a single point calculation with b3lyp-D3BJ/ma-TZVPP, corresponding to the method and basis set used by Lu et al. in their predictive model (Equation (2)) [21].

(2) $$\Delta E\left(\mathrm{kcal}/\mathrm{mol}\right)=-223.08\;\rho \left(r_{\mathrm{BCP}}\right)+0.7423$$

Here, the mean absolute percentage error of prediction (MAPE) is 14.7% and $\rho \left(r_{\mathrm{BCP}}\right)$ is the electron density at the critical bond point of the corresponding interaction, determined by the Multiwfn program (Version 3.8) [58].

### 3.4. Calculation of the Complex Formation Interaction Energies (ΔΕ_int_)

The interaction energies of the host-guest complexes and host-host dimers were calculated by the total electronic energy difference between the complex and the isolated constituting building blocks. The electronic structure of each building block was the same individually as in the supramolecular complex. The basis set superposition error (BSSE) of the complexes was calculated and considered in the correction of the total electronic energy. All energies described were determined using the B97-3c level of theory [54], implemented in the ORCA program package (Program Version 5.0.3) [55].

### 3.5. Calculation of the Complex Formation Interaction Energies Based on Molecular Forcefield (ΔΕ_int-FF_)

The ΔΕ_int-FF_ was calculated as the sum of the electrostatic interaction energy (ΔΕ_ele_) and the vdW interaction energy (ΔΕ_vdW_) using the potential of pairwise non-covalent interactions (Equations (3) and (4)) [61].

(3) $${\Delta E}_{\mathrm{ele}}=\frac{1}{{4\pi \varepsilon }_{0}}\underset{a\;\epsilon \;A}{\sum }\underset{\;b\;\epsilon \;B}{\sum }\frac{q_{a}q_{b}}{r_{\mathrm{ab}}}$$

$q_{a}$ and $q_{b}$ are the partial charges centred on the individual atoms of the building blocks A and B, respectively. $r_{\mathrm{ab}}$ is the separation distance and $\varepsilon_{0}$ the vacuum permittivity. The MK charges were calculated [57] and used as partial charges in each building block, with the exact conformation that they individually possess in the supramolecular complex through a single point energy calculation at the B97-3c level of theory.

(4) $${\Delta E}_{\mathrm{vdW}}=\underset{a\;\epsilon A\;}{\sum }\underset{b\;\epsilon B}{\sum }{4\varepsilon }_{\mathrm{ab}}\left[{\left(\frac{\sigma_{\mathrm{ab}}}{r_{\mathrm{ab}}}\right)}^{12}-\;{\left(\frac{\sigma_{\mathrm{ab}}}{r_{\mathrm{ab}}}\right)}^{6}\right]$$

The parameters $\varepsilon_{\mathrm{ab}}$ and $\sigma_{\mathrm{ab}}$ [62], defined as $\varepsilon_{\mathrm{ab}}=\sqrt{\varepsilon_{a}{+\varepsilon }_{b}\;}$ and $\sigma_{\mathrm{ab}}{=\sigma }_{a}{+\sigma }_{b}$, were obtained using GAFF and AMBER99 force fields, using the molecular structure of the complex obtained by DFT (as discussed above). Equations (3) and (4) were calculated by the Multiwfn program [58].

### 3.6. Calculation of the Complex Formation Free Binding Energies (ΔG_calc_)

The free binding energy for the formation of the complex was estimated from Equation (5) [63].

(5) $$\Delta G_{\mathrm{calc}}=\Delta G_{\mathrm{aq}}\left(\mathrm{complex}\;\mathrm{AB}\right)\;-\;\Delta G_{\mathrm{aq}}\left(A\right)\;-\;\Delta G_{\mathrm{aq}}\left(B\right)\;$$

The free binding energies of the building blocks A and B, as well as of the complex AB were calculated in the aqueous phase (ΔG_aq_) using the B97-3c level of theory at 298.15 K and 1 atm from the ORCA program, with previous optimization of their structures assuming implicit solvent (water). The solvation was simulated using the conductor-like polarizable continuum model with the COSMO epsilon function (CPCMC) [64]. Vibrational entropy was computed according to the quasi-rigid-rotor harmonic oscillator (QRRHO) approximation [65].

## 4. Conclusions

With this work, we characterize the non-covalent interactions of **β-CD** and **DM-β-CD** host-guest complexes, and more precisely the intramolecular interactions within the hosts, the intermolecular host-guest interactions with phenol as well as the host dimer interactions. In support of experimental data, our results suggest significantly more stable host-guest complexes of the methylated host **DM-β-CD** with phenol compared to **β-CD**. We attribute this behaviour to the disruption of the HB network in the primary face of **β-CD** upon methylation, which leads to a greater flexibility, and hence a larger stabilization of the guest through both vdW interactions and stronger intermolecular HBs.

For the intramolecular interactions in the CDs, free **β-CD** presented a network of strong hydrogen bonds with an average energy of −5.66 kcal/mol on the primary face and −5.86 kcal/mol on the secondary face. In contrast, the free **DM-β-CD** yielded lower and more fluctuating energies, attributable to the loss of symmetry in the CD portals (due to electronic repulsions upon methylation) and the absence of intramolecular HBs in the primary face.

The lack of the latter in **DM-β-CD** allows on the one hand -OCH_3_ groups to form stronger intermolecular interactions with hydrogen bond donor groups such as the -OH group of phenol (OH-OCH_3_, ≈1.5-fold relative to the hydroxyl groups of **β-CD**), and on the other hand leads to a greater flexibility with respect to the guest, where stronger intermolecular vdW interactions occur (≈1.3-fold).

When investigating the dimerization of both CD hosts, we found a greater stability of the **β-CD** dimer compared to **DM-β-CD**. Although this is not directly related to the observed stability of the CD-phenol complexes on a molecular level as discussed above, the greater stability of the **β-CD** dimer over the **DM-β-CD** dimer suggests that if these dimers are formed in such host-guest assays, the binding constant in the presence of **β-CD** will be underestimated to a greater extent than the constant obtained in the presence of **DM-β-CD**.

Taken together, the applied methodology presents a complementary way of assessing non-covalent interactions in host-guest complexes, and we propose this theoretical framework as a suitable addition to the analytical toolkit of such supramolecular structures.

## Figures and Tables

**Table 1:** Intramolecular interaction energies (in kcal/mol) within **β-CD** (left) and **DM-β-CD** (right), calculated from the electron density at the critical bond points. The standard error of the mean was calculated for both faces of both hosts.

Monomer	β-CD		DM-β-CD	
	Primary Face	Secondary Face	Primary Face	Secondary Face
	ΔE (HB)	ΔE (HB)	ΔE (vdW)	ΔE (HB)
**1**	−5.76	−5.69	−0.06	−4.12
**2**	−5.71	−5.79	−0.02	−4.18
**3**	−5.45	−5.94	−0.22	−4.29
**4**	−5.83	−5.99	−0.22	−5.57
**5**	−5.72	−5.78	−0.19	−3.79
**6**	−5.58	−5.81	−0.04	−3.07
**7**	−5.54	−6.02	−0.18	−5.13
**Average**	−5.66 ± 0.05	−5.86 ± 0.04	−0.13 ± 0.03	−4.31 ± 0.29

**Table 2:** Binding parameters of CD/phenol host-guest complexes (in kcal/mol).

Binding Parameters	β-CD/Phenol	DM-β-CD/Phenol
Averaged individual intermolecular interaction energy	−4.99 ^a^	−7.31 ^a^
Averaged individual intramolecular interaction energy ^c^	−5.38 ^a^	0.17 ^b^
Interaction energy (ΔΕ_int_)	−14.96	−18.27
Interaction energy based on molecular forcefield (ΔΕ_int-FF_)	−15.94	−20.84
Electrostatic interaction energy (ΔΕ_ele_)	−4.55	−6.17
vdW interaction energy (ΔΕ_vdW_)	−11.40	−14.67
Estimated free binding energy (ΔG_calc_)	−2.62	−5.23
Experimental free binding energy (ΔG_exp_)	−2.69 ^d^	NF ^e^

^a^ Hydrogen bond interaction at the primary face. ^b^ vdW interaction at the primary face. ^c^ Intramolecular interaction from the oxygen that partakes in the intermolecular interaction with phenol. ^d^ Experimental data from [26]. ^e^ NF: experimental data not found.

**Table 3:** Binding parameters for the host-host dimers of **β-CD** and **DM-β-CD** in kcal/mol.

Binding Parameters	β-CD Dimer	DM-β-CD Dimer
Averaged individual intermolecular interaction energy	−5.33 ^a^	ND ^b^
Averaged individual intramolecular interaction energy	−5.22 ^a^	ND ^b^
Interaction energy (ΔΕ_int_)	−53.88	−41.65
Interaction energy based on molecular forcefield (ΔΕ_int-FF_)	−61.16	−58.77
Electrostatic interaction energy (ΔΕ_ele_)	−46.40	−13.28
vdW interaction energy (ΔΕ_vdW_)	−14.75	−45.48
Estimated free binding energy (ΔG_calc_)	−12.84	ND ^b^

^a^ Hydrogen bond interaction at the primary face. ^b^ ND: not determined.

## Data Availability

The supporting data referred to in this manuscript is contained within a Supplementary Information Document and in a Supplementary Dataset available on Figshare 10.6084/m9.figshare.22010837.

## Supplementary Materials

The following supporting information can be downloaded at: https://www.mdpi.com/article/10.3390/molecules28062625/s1. Color-filled scatter plots of the reduced density gradient versus the sign of the second Hessian eigenvalue multiplied by the electron density for **β-CD, DM-β-CD**, the **β-CD** dimer, the **DM-β-CD** dimer, the **β-CD**/phenol complex and the **DM-β-CD**/phenol complex; including the description of the color scale.
