# Supplementary material for: Influence of β-Cyclodextrin Methylation on Host-Guest Complex Stability: A Theoretical Study of Intra- and Intermolecular Interactions as Well as Host Dimer Formation
Source: Molecules. 2023 Mar 14;28(6):2625. doi: 10.3390/molecules28062625 (PMC10054123; doi:10.3390/molecules28062625)
Supplement: Supplementary file 1 [file molecules-28-02625-s001.zip › molecules-2231837-supplementary.pdf]

Supporting Information

Influence of β-Cyclodextrin Methylation on Host-Guest Complex Stability: A Theoretical Study of Intra- and Intermolecular Interactions as well as Host Dimer Formation

Niklas Geue[1], Jackson J. Alcázar[2,*] and Paola R. Campodónico[2]

[1]Michael Barber Centre for Collaborative Mass Spectrometry, Manchester Institute of Biotechnology, Department of Chemistry, The University of Manchester, 131 Princess Street, Manchester, M1 7DN, UK. [2]Centro de Química Médica, Facultad de Medicina Clínica Alemana, Universidad del Desarrollo, Santiago 7780272, Chile.

[*]Corresponding Author: jackson.alcazar@udd.cl

Table of Contents:

Figure S1. Color-filled scatter plots of the reduced density gradient versus the sign of the second Hessian eigenvalue multiplied by the electron density of a) **β-CD** and b) **DM- β-CD**. The data was obtained by evaluating the B97-3c density. To cover the entire system, the grid configuration used was about 1728000 points in total. c) Description of the color scale according to sign(λ_2)ρ, λ_2 and ρ. 3

Figure S2. Color-filled scatter plots of the reduced density gradient versus the sign of the second Hessian eigenvalue, multiplied by the electron density of a) the **β-CD dimer**, b) the **DM-β-CD dimer**, c) the **β-CD/phenol** complex and d) the **DM-β-CD/phenol** complex. The data was obtained by evaluating the B97-3c density. To cover the entire system, the grid configuration used was around 1728000 points in total. 4

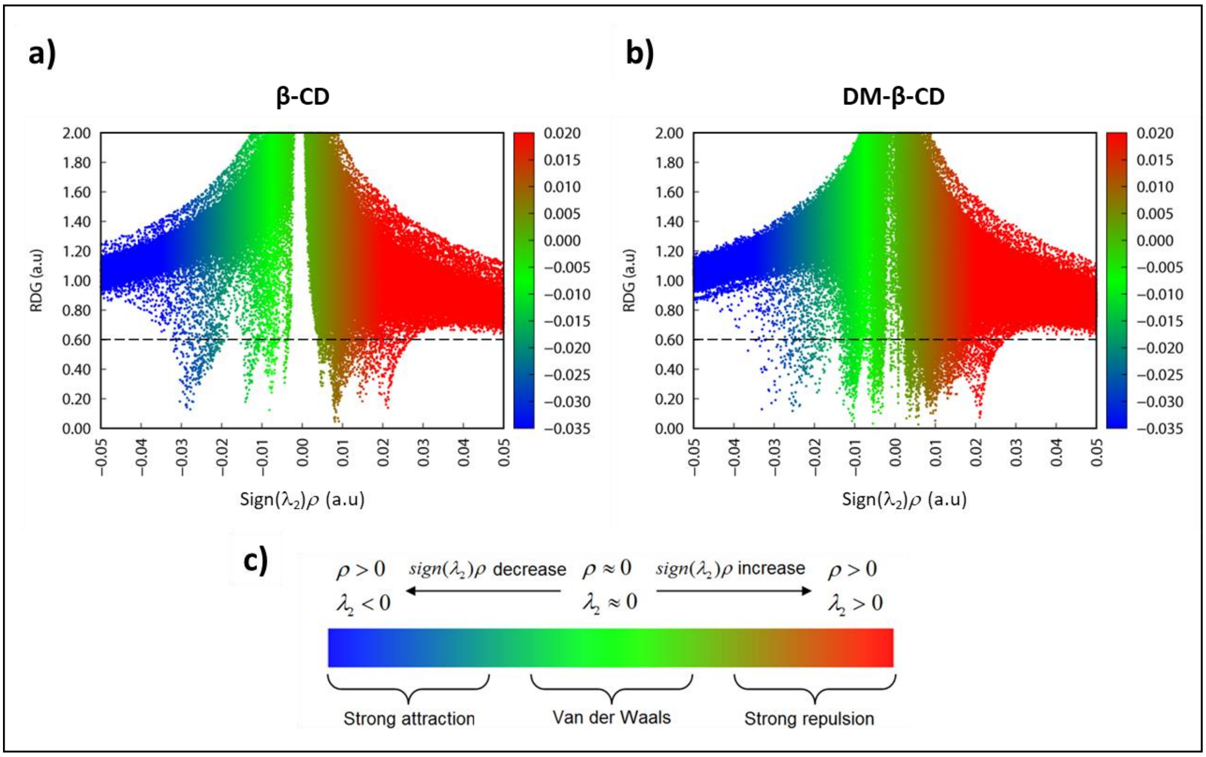

Figure S1. Color-filled scatter plots of the reduced density gradient versus the sign of the second Hessian eigenvalue multiplied by the electron density of a) **β-CD** and b) **DM- β-CD**. The data was obtained by evaluating the B97-3c density. To cover the entire system, the grid configuration used was about 1728000 points in total. c) Description of the color scale according to sign(λ_2)ρ, λ_2 and ρ.

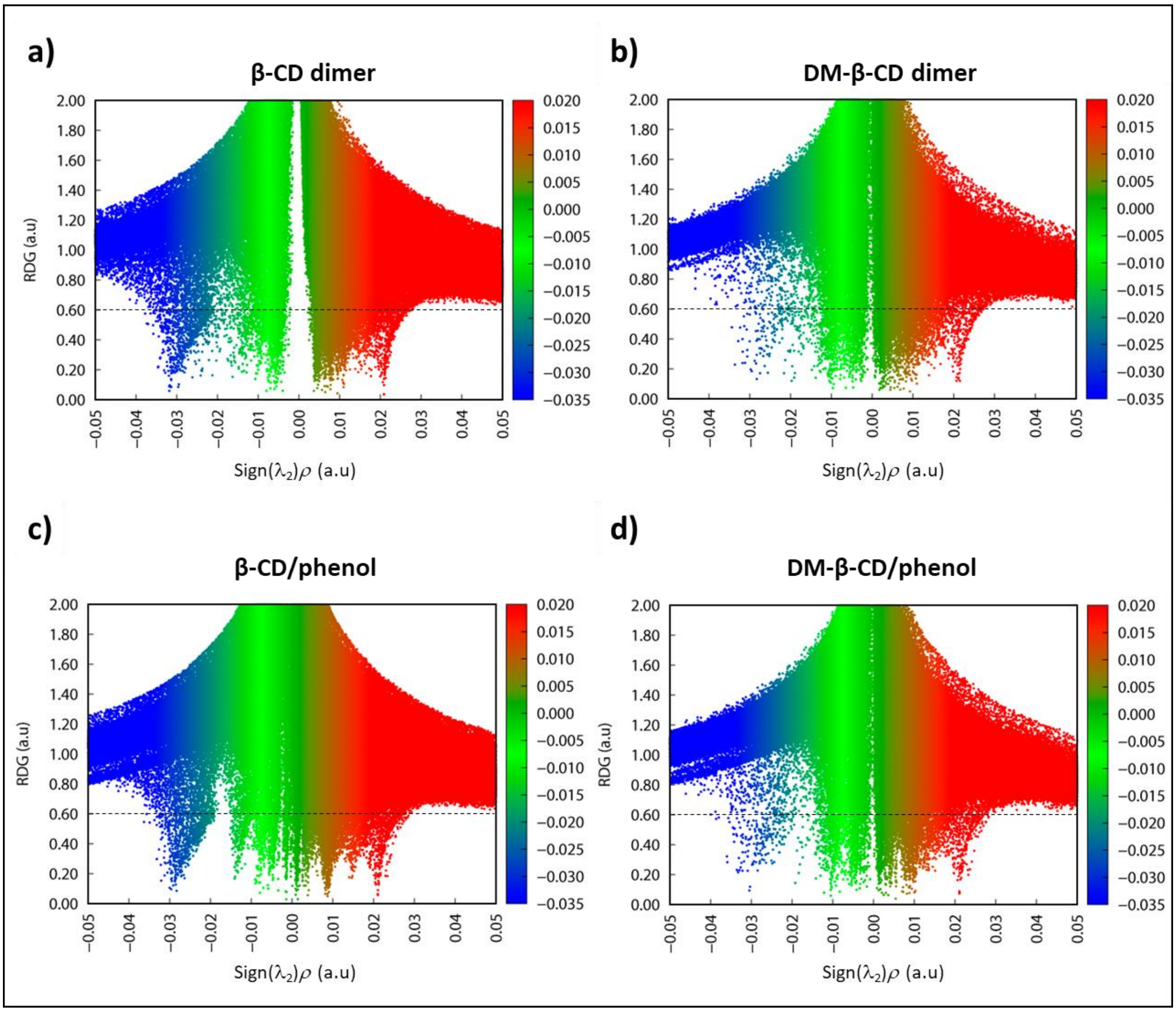

Figure S2. Color-filled scatter plots of the reduced density gradient versus the sign of the second Hessian eigenvalue, multiplied by the electron density of a) the **β-CD dimer**, b) the **DM-β-CD dimer**, c) the **β-CD/phenol** complex and d) the **DM-β-CD/phenol** complex. The data was obtained by evaluating the B97-3c density. To cover the entire system, the grid configuration used was around 1728000 points in total.
